# Book Review

**Published:** 1954-07

**Authors:** 


### Book Review

"ARTHUR RENDLE SHORT", by W. M. CAPPER and D. JOHNSON. Published by the Inter-Varsity Fellowship at 8s. 6d.

Many old students of Bristol and others who knew Rendle Short will wish to possess this book. The writing of it was obviously a labour of love for the authors and they have admirably discharged their debt to a great Christian teacher of Medicine. In addition they have retold many excellent stories which he used to give point and interest to his teaching.

